# Pre-diagnostic anthropometry, sex, and risk of colorectal cancer according to tumor immune cell composition

**DOI:** 10.1080/2162402X.2019.1664275

**Published:** 2019-09-19

**Authors:** Jonna Berntsson, Jakob Eberhard, Björn Nodin, Karin Leandersson, Anna H Larsson, Karin Jirström

**Affiliations:** a Department of Clinical Sciences Lund, Oncology and Pathology, Lund University, Lund, Sweden; b Cancer Immunology, Department of Translational Medicine, Lund University, Malmö, Sweden

**Keywords:** Obesity, sex, anthropometry, colorectal cancer, immune microenvironment, PD-L1

## Abstract

Obesity is a well-established risk factor for colorectal cancer (CRC), but the association with the tumor microenvironment has been sparsely described. Herein, we examined the relationship between pre-diagnostic anthropometry and CRC risk according to tumor immune cell composition, with particular reference to potential sex differences.

The density of different immune cell subsets was assessed by immunohistochemistry in tissue microarrays with tumors from 584 incident CRC cases in a prospective, population-based cohort (n = 28098). Multivariable Cox regression models, adjusted for age, smoking, alcohol intake, and educational level, were applied to calculate risk of immune marker-defined CRC in relation to quartiles of pre-diagnostic height, weight, body mass index (BMI), waist and hip circumferences, waist-hip ratio (WHR), and body fat percentage (BFP).

Obesity was all over significantly associated with risk of CRC with low density of FoxP3^+^ T cells and low programmed cell-death protein 1 (PD-L1) expression on tumor cells, but with high density of CD8^+^ T cells and CD20^+^ B cells. In women, obesity was significantly associated with risk of PD-L1 high tumors (p= 0.009 for weight, p= 0.039 for BMI). Contrastingly, in men, obesity defined by all anthropometric factors was significantly associated with PD-L1 low tumors (p= 0.005 for weight, p = 0.002 for BMI, p<0.001 for waist, p= 0.011 for hip, p<0.001 for WHR, and p= 0.004 for BFP).

In summary, obesity appears to influence the immune landscape of CRC, possibly in a sex-dependent manner. Thus, anthropometry and sex may be important factors to take into account when assessing the prognostic or predictive value of relevant complementary immune biomarkers.

## Introduction

A plethora of studies have examined the relationship between body weight and risk of colorectal cancer (CRC), mainly demonstrating a positive association between high weight or body mass index (BMI) and an increased risk of CRC.^1–3^ However, this association mainly seems to apply to colon cancer,^1^ and the relative risk is higher in men than in women.^4^ Although obesity-associated low-grade chronic inflammation is a widely accepted factor in CRC carcinogenesis,^5^ the underlying pathophysiological mechanisms remain unclear.

The role of the immune system in regulating CRC development is becoming increasingly recognized^6^ and evidence suggests influences of both exogenous and endogenous factors on tumor-immune interactions, indicating the need for integrative analyses.^7^ The associations between the density of different immune cell subsets and disease stage, grade, tumor location, and mutation status have been investigated in numerous studies,^6,8^ also in the herein investigated cohort.^9–11^ However, the relationship with obesity has been sparsely described.

One experimental study demonstrated an impaired function in natural killer cells in obese rats with CRC.^12^ Furthermore, muscle depletion in surgically treated CRC patients has been found to be associated with dendritic cells with lower co-stimulatory and migratory capacities.^13^ To the best of our knowledge, only two studies have thus far examined the immune tumor microenvironment in CRC with reference to obesity, the first finding no significant differences between BMI and CRC incidence according to tumor lymphocyte infiltration.^14^ However, a recent study across multiple tumor models demonstrated fewer CD3^+^ T cells in obese CRC patients, and the authors also suggested that obesity might be predictive of immune checkpoint blockade.^15^ Nevertheless, these studies only considered T cell infiltration, and BMI was the only anthropometric factor used to define obesity. Moreover, no previous studies have hitherto investigated the relationship between obesity and the density of B lymphocytes or expression of programmed cell-death ligand 1 (PD-L1) on immune cells and tumor cells in CRC.

In this study, with data from a large prospective population-based cohort, we examined the relationship between pre-diagnostic obesity, measured as several anthropometric factors, and CRC risk according to the density of tumor-infiltrating immune cells of the T lineage (CD8^+^ and FoxP3^+ ^cells) and B lineage (CD20^+ ^cells), as well as PD-L1 expression on immune cells and tumor cells.

## Results

### Baseline characteristics

The distribution of well-established risk factors in CRC cases and in the rest of the cohort is shown in Table 1. Compared to the rest of the cohort, CRC cases were slightly older (*p* < .001 for both sexes), had a higher daily alcohol consumption (*p* = .041 for men and *p* = .005 for women), had higher weight (*p* = .010 for men and *p* < .001 for women) and BMI (*p* < .001 for both sexes), had an increased waist circumference (*p* < .001 for both sexes) and hip circumference (*p* < .001 for women), a higher waist-hip ratio (WHR) (*p* < .001 for both sexes), and a higher body fat percentage (BFP) (*p* < .001 for both sexes). Smoking status did not differ between cases and non-cases.

**Table 1.** Distribution of risk factors in cases and rest of the cohort.

Factor	Cases (*n* = 584)	Rest of cohort (*n* = 27340)	*p*	Cases, men (*n* = 280)	Rest of cohort, men (*n* = 10703)	*p*	Cases, women (*n* = 304)	Rest of cohort, women (*n* = 16637)	*p*
Age at baseline	61.9 (6.8)	57.6 (7.6)	*<0.001*	61.7 (6.7)	59.1 (7.0)	*<0.001*	62.1 (6.9)	57.3 (7.9)	*<0.001*
Height, cm	169.5 (8.8)	168.0 (8.8)	*0.002*	176.3 (6.4)	176.4 (6.6)	*<0.001*	163.3 (5.5)	163.6 (6.1)	*<0.001*
Weight, kg	76.6 (14.2)	72.0 (13.6)	*<0.001*	83.8 (12.7)	81.7 (12.1)	*0.010*	70.0 (12.2)	68.0 (11.7)	*<0.001*
Body-mass index, kg/m^2^	26.6 (4.0)	25.3 (4.0)	*<0.001*	26.9 (3.6)	26.2 (3.5)	*<0.001*	26.3 (4.4)	25.4 (4.2)	*<0.001*
Waist circumference, cm	87.9 (13.7)	83.0 (14.5)	*<0.001*	96.3 (10.7)	93.7 (12.6)	*<0.001*	80.1 (11.3)	77.8 (12.1)	*<0.001*
Hip circumference, cm	100.7 (9.0)	98.0 (8.8)	*<0.001*	101.2 (7.4)	99.2 (7.1)	*0.055*	100.2 (10.2)	97.8 (9.8)	*<0.001*
Waist-hip-ratio	0.87 (0.10)	0.84 (0.14)	*<0.001*	0.95 (0.06)	0.94 (0.10)	*<0.001*	0.80 (0.05)	0.80 (0.14)	*<0.001*
Body-fat percentage, %	26.9 (7.1)	27.0 (7.0)	*0.023*	21.5 (5.0)	20.7 (5.0)	*<0.001*	31.8 (4.9)	30.7 (5.0)	*<0.001*
Alcohol consumption, g/day	10.8 (14.2)	7.2 (12.7)	*0.005*	15.7 (17.4)	15.5 (16.0)	*0.041*	6.2 (8.1)	7.7 (8.7)	*0.005*
Smoking status									
Regularly	124 (21.2)	6521 (23.9)		53 (18.9)	2562 (23.9)		71 (23.4)	3959 (23.8)	
Occasionally	20 (3.4)	1237 (4.5)		12 (4.3)	519 (4.8)		8 (2.6)	718 (4.3)	
Former smoker	232 (39.7)	9211 (33.7)		145 (51.8)	4595 (42.9)		87 (28.6)	4616 (27.7)	
Never smoked	208 (35.6)	10359 (37.9)	*0.194*	70 (25.0)	3021 (28.2)	*0.071*	138 (45.4)	7338 (44.2)	*0.780*

Median [standard deviation (SD)] presented for continuous variables.

There were 52.1% women and 47.9% men among cases. Women had a significantly lower alcohol consumption than men (*p* < .001); however, there were no other significant differences in the distribution of baseline characteristics, excluding the anthropometric factors, or the clinicopathological characteristics between men and women (Supplementary Table 1). Furthermore, the distribution of baseline characteristics and clinicopathological characteristics in CRC cases in the extended cohort (n = 626), including non-EPIC (European Prospective Investigation into Cancer) cases, did not differ significantly from the CRC cases included in the EPIC cohort (n = 584) (Supplementary Table 2).

### Relationship between pre-diagnostic anthropometry and colorectal cancer risk according to tumor immune cell composition

**Figure 1.**
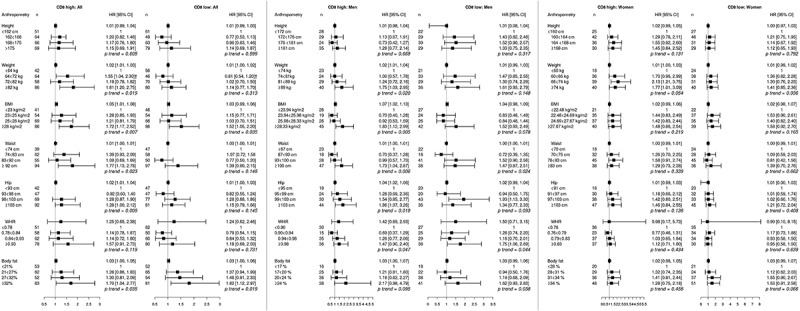
Adjusted hazard ratios (HR) and 95% confidence intervals (CI) for colorectal cancer risk in relation to anthropometric measurements, defined as continuous variables as well as quartiles, and CD8^+^ T cell infiltration, in the full cohort, in men, and in women. Cases were divided into groups of high (≥134, n = 250) and low (<134, n = 252) CD8^+^ T cell count. The multivariable Cox regression model includes age, smoking, alcohol intake, and educational level. †Heterogeneity analysis with *p* < .05.

**Figure 2.**
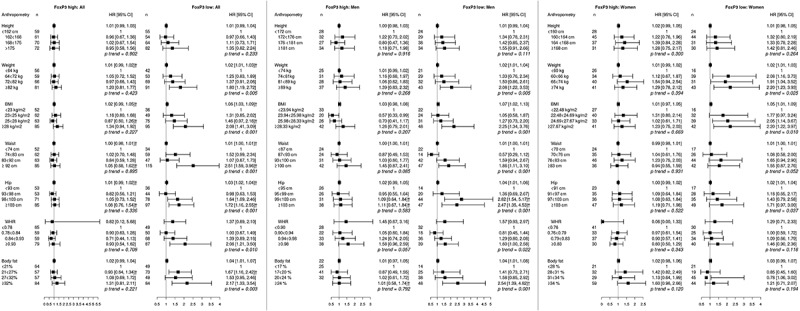
Adjusted hazard ratios (HR) and 95% confidence intervals (CI) for colorectal cancer risk in relation to anthropometric measurements, defined as continuous variables as well as quartiles, and FoxP3^+^ T cell infiltration, in the full cohort, in men, and in women. Cases were divided into groups of high (≥8, n = 262) and low (<8, n = 255) FoxP3^+^ cell count. The multivariable Cox regression model includes age, smoking, alcohol intake, and educational level. †Heterogeneity analysis with *p* < .05.

**Figure 3.**
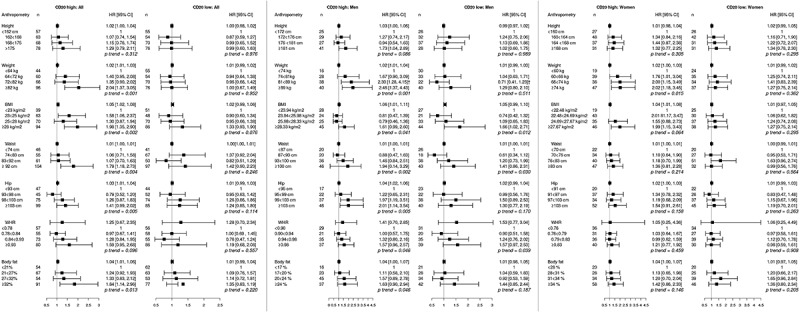
Adjusted hazard ratios (HR) and 95% confidence intervals (CI) for colorectal cancer risk in relation to anthropometric measurements, defined as continuous variables as well as quartiles, and CD20^+^ B cell infiltration, in the full cohort, in men, and in women. Cases were divided into groups of high (≥3, n = 266) and low (<3, n = 255) CD20^+^ B cell count. The multivariable Cox regression model includes age, smoking, alcohol intake, and educational level. †Heterogeneity analysis with *p* < .05.

**Figure 4.**
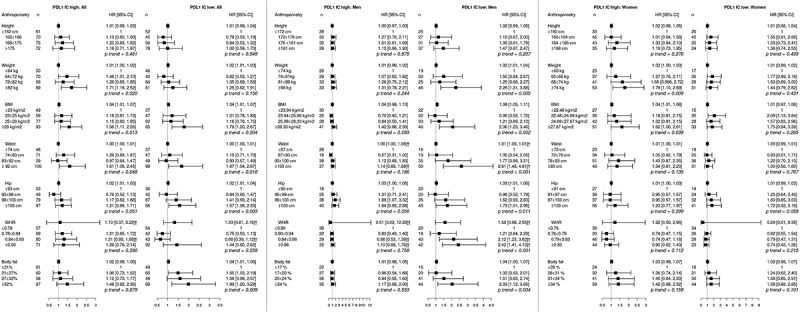
Adjusted hazard ratios (HR) and 95% confidence intervals (CI) for colorectal cancer risk in relation to anthropometric measurements, defined as continuous variables as well as quartiles, and programmed cell-death ligand 1 (PD-L1) expression on immune cells, in the full cohort, in men, and in women. Cases were divided into groups of high (n = 278) and low (n = 231) expression, according to a cutoff at 10%. The multivariable Cox regression model includes age, smoking, alcohol intake, and educational level. †Heterogeneity analysis with *p* < .05.

**Figure 5.**
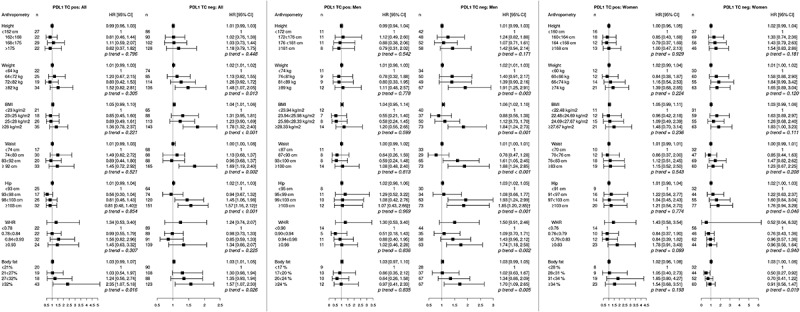
Adjusted hazard ratios (HR) and 95% confidence intervals (CI) for colorectal cancer risk in relation to anthropometric measurements, defined as continuous variables as well as quartiles, and programmed cell-death ligand 1 (PD-L1) expression on tumor cells, in the full cohort, in men, and in women. Cases were divided into groups of high (n = 100) and low (n = 409) expression, according to a cutoff at 10%. The multivariable Cox regression model includes age, smoking, alcohol intake, and educational level. †Heterogeneity analysis with *p* < .05.

The relationship between pre-diagnostic anthropometry, defined as continuous variables as well as quartiles, and CRC risk according to low and high expression of different tumor-infiltrating immune cells, and PD-L1 expression on tumor cells, overall and in men and in women, respectively, are shown in Figure 1–5. Statistically significant heterogeneities are marked in all figures. There were no significant differences to the results when excluding cases with tumor material available only from biopsies (data not shown).

The time-dependent covariate was non-significant for all investigated immune cells as well as PD-L1 expression on immune cells and on tumor cells, and therefore, the factor x time interaction term was dropped from the model. The proportional hazards assumption was also considered to be satisfied with graphical evaluation using log-minus-log plots (data not shown).

Hazard ratios for CRC risk according to CD8^+^ T cell density are shown in Figure 1. Overall, several anthropometric factors were associated with CD8^+^ high tumors, with the highest risks observed in the top quartiles of weight, BFP, waist, hip, and BMI. Significant risks of CD8 low tumors were also observed for the top quartiles of BFP and BMI. In sex-stratified analyses, the significant associations between obesity and CD8 high tumors were mainly retained in men, and high WHR was significantly associated with CD8 low tumors. In women, a significant risk of CD8 high tumors was only observed for the 3^rd^ and 4^th^ quartiles of weight, with a borderline significant trend, and none of the anthropometric factors were associated with risk of CD8 low tumors. There was no significant interaction between sex and risk of CRC defined by CD8^+^ T cell density.

Hazard ratios for CRC risk according to FoxP3^+^ T cell density are shown in Figure 2. Overall, all anthropometric factors except height were significantly associated with FoxP3 low tumors, with the highest risks observed in the top quartiles, confirmed in the heterogeneity analysis for all factors except WHR. None of the anthropometric factors were associated with FoxP3 high tumors. In men, all associations between obesity and FoxP3 low tumors, except for WHR, were retained, and further confirmed in the test for heterogeneity for hip circumference and BFP. In women, significant trends for associations of obesity with FoxP3 low tumors were observed for weight, hip and BMI. There was no significant interaction between sex and risk of CRC defined by FoxP3^+^ T cell density.

Hazard ratios for CRC risk according to CD20^+^ B cell density are shown in Figure 3. Overall, all anthropometric factors except height and WHR were significantly associated with CD20 high tumors, with the highest risks observed in the top quartiles. In men, all associations between obesity and CD20 high tumors were retained, and further confirmed in the test for heterogeneity for weight. In women, only high weight was significantly associated with risk of CD20 low tumors. There was no significant interaction between sex and risk of CRC defined by CD20^+^ B cell density.

Hazard ratios for CRC risk according to PD-L1 expression on immune cells are shown in Figure 4. Overall, several anthropometric measurements of obesity were significantly associated with risk of tumors displaying high or low PD-L1 expression on immune cells, confirmed in heterogeneity analysis for WHR. High waist circumference and BMI were associated with both PD-L1 high and PD-L1 low tumors. In men, all anthropometric factors, except height, were significantly associated with risk of tumors displaying low PD-L1 expression on immune cells, confirmed in heterogeneity analysis for waist (both as continuous variable and in quartiles) and WHR (both as continuous variable and in quartiles). Contrastingly, in women, high weight and BMI were associated with risk of tumors displaying high PD-L1 expression on immune cells. There was no significant interaction between sex and risk of CRC defined by PD-L1 expression on immune cells.

Hazard ratios for CRC risk according to PD-L1 expression on tumor cells are shown in Figure 5. Overall, all factors except height and WHR were significantly associated with risk of tumors displaying negative tumor-cell specific PD-L1 expression, confirmed in heterogeneity analysis for hip circumference, and BFP was associated with tumors displaying both positive and negative tumor-cell specific PD-L1 expression. In men, all anthropometric factors, except height, were significantly associated with risk of CRC with negative tumor-cell specific PD-L1 expression, confirmed in heterogeneity analysis for hip circumference. In women, only hip circumference and BFP were significantly associated with risk of CRC with negative tumor-cell specific PD-L1 expression. There was no significant interaction between sex and risk of CRC defined by PD-L1 expression on tumor cells.

Similar results were also obtained for risk of CRC according to T and B cell density when using the previously defined prognostic cutoffs derived from classification and regression tree^10,11^ (data not shown).

## Discussion

In this large, prospective cohort study, we have investigated the relationship between obesity and risk of CRC subtype defined by the character of the tumor immune microenvironment, with particular reference to potential difference between sexes. As the hitherto first study to investigate the impact of obesity on the expression of PD-L1 in CRC, the results demonstrated that several anthropometric factors were associated with risk tumors with of low immune cell-specific PD-L1 expression in men, but with risk of tumors with high immune cell-specific PD-L1 expression in women. Furthermore, significant associations were found between obesity and risk of tumors with low infiltration of FoxP3^+^ T cells, but with risk of tumors with high infiltration of CD8^+^ T cells and CD20^+^ B cells.

A previous study reported significantly fewer tumor-infiltrating CD3^+^ T cells in obese CRC patients,^15^ However, this study only investigated the density of T cells in relation to BMI, and did not consider potential sex differences. The herein demonstrated associations between obesity and risk of tumors with high density of CD8^+^ cytotoxic lymphocytes, in particular in men, but with low density of FoxP3^+^ regulatory T-cells, suggest a differential relationship of obesity with different subsets of T lymphocytes. Hence, the use of a pan-T cell marker will only provide a rough assessment of the link between obesity and the immune microenvironment of CRC defined by cells of the T lineage. The findings in the present study highlight the need for more refined analyses of T lymphocyte subsets in order to better assess potential associations between obesity and the composition of the immune microenvironment in CRC. Hanyuda et al. indeed investigated the relationship between BMI and risk of CRC, defined by the levels of CD3^+^, CD8^+^, FoxP3^+^, and CD45RO^+^ T cells, but found no heterogeneous associations.^14^ Potential sex differences were, however, not considered. Previous studies have demonstrated that high BMI in men is more closely related to central or abdominal obesity, whereas high BMI in women mostly correlates with lower body obesity.^3^ Epidemiological studies have demonstrated both increased weight and waist circumference, although self-reported, being associated with an increased risk of CRC in both men and women,^3^ and thus, it has been hypothesized that body fat distribution is more important than weight or BMI in women. In the present study, we considered seven different anthropometric measurements, and found several differences between men and women regarding the associations between the anthropometric factors and the risk of CRC with different immune cell infiltrations.

To the best of our knowledge, this is the first study to investigate the impact of obesity on CRC risk defined by B cell infiltration. The results demonstrated that all anthropometric factors indicative of obesity were significantly associated with risk of CRC with high B cell density, and that these associations were particularly evident in men. In the herein investigated cohort, we have previously found high B cell density to be an independent favorable prognostic factor.^10^ Previous studies have demonstrated that B cells are required for optimal cell-mediated anti-tumor immunity,^16^ and in ovarian cancer, CD20^+^ B cells have been suggested to support CD8^+^ T cells in tumor immune response.^17^ Collectively, the findings from the present study suggest that pre-diagnostic obesity promotes CRC with a more favorable tumor immune microenvironment, defined by high infiltration of CD8^+^ T cells and CD20^+^ B cells. Moreover, a possible effect of sex was observed in that the associations were more pronounced in men. However, as there were no significant interactions between sex and anthropometric factors in relation to risk of immune marker-defined CRC, no firm conclusions can be drawn and these observations need to be confirmed in additional studies.

Although obesity is a well-known risk factor for several types of cancer, including CRC, previous studies have demonstrated an improved survival for overweight patients compared with normal-weight patients.^18^ Sarcopenic obesity, however, i.e. simultaneous obesity and low muscle mass, represents a clinically different body composition type, with significantly poorer prognosis.^19^ This “obesity paradox” has been attributed to associations with a less aggressive tumor phenotype, although confounding factors have also been suggested, e.g. the use of BMI as beforementioned, additional nutrient reserve in adipose tissue, or a different chemotherapy metabolism, reducing overall toxicity.^20^ Interestingly, obesity has also been found to increase T cell aging, resulting in higher PD-1 expression and dysfunction in animal models as well as in humans.^15^ This dysfunction may be exploited in immune checkpoint blockade, possibly explaining results from recent clinical studies demonstrating an improved response to PD-1/PD-L1 blockade in obese patients.^21^ Moreover, a recent meta-analysis reported a greater benefit of PD-1 or PD-L1 blockade in men than in women,^22^ and studies have demonstrated sex hormones to modulate the expression and function of PD-1 and PD-L1.^23,24^ Our findings demonstrate contrasting associations between sexes regarding obesity and risk of CRC defined by expression of PD-L1 on immune cells. Although there was no significant interaction, these findings suggest an integrative influence of both sex and anthropometric factors in shaping anti-tumor immune response. In several types of cancer, PD-L1 expression is used as a predictive biomarker for immune checkpoint inhibitors; however, in CRC, deficient mismatch repair/microsatellite instability (dMMR/MSI) status is considered a superior predictor of treatment response than levels of PD-L1 expression. In this regard, previous studies, including on the present cohort, have demonstrated that obesity is associated with an increased risk of mismatch repair proficient/microsatellite stable (pMMR/MSS) tumors in both sexes,^25,26^ whereas another study found a link of obesity and risk of tumors displaying dMMR/MSI tumors only in women .^27^ Although no association was found between obesity and MSI in a previous study on the present cohort,^26^ immune infiltration and PD-L1 expression on immune cells have been found to be significantly higher in dMMR tumors,^9–11^ which is in line with the expected.^28^

Colorectal cancer is a heterogeneous disease, with multiple differences between proximal and distal tumors, including immune cell infiltration.^29^ Although not investigated herein, due to the small numbers in each subgroup, future studies should assess the association between obesity and risk of CRC with particular reference to the primary tumor location.

Several studies regarding the impact of obesity on CRC risk have used self-reported anthropometric measurements. In the present study, all anthropometric data were collected by trained nurses. Although there is a potential risk for inter-observer variation, there were strict recommendations for how measurements should be made and we, therefore, consider the risk of misclassification to be low. However, as anthropometric data only were obtained at baseline, there is a possibility of weight gain or loss for some individuals, which may lead to misclassification of risk.

Our study also possesses several limitations due to its observational design. Firstly, a potential selection bias might arise when excluding cases with insufficient tumor material, although the distribution of each risk factor and clinicopathological characteristics in included cases did not considerably differ from excluded cases. Secondly, there is also a potential bias in that participation in the Malmö Diet and Cancer Study (MDCS) might *per se* be associated with a certain body constitution. However, a previous study regarding the representativeness of the MDCS found an equal distribution of BMI in participants and in the background population.^30^ Thirdly, there is a risk of type II errors due to the relatively small number of cases in some groups. For example, the number of cases in the analyses related to CD8 low is generally low. However, when using the median value as cutoff, with more equally sized groups, the results did not differ. Nonetheless, these results must be taken with precaution. Finally, as multiple testing was performed, there is a risk of type I error. To address this issue, it has been recommended to perform a heterogeneity test,^31^ as was done in the present study. The herein used cutoffs to define high and low immune cell infiltration and PD-L1 expression should also be discussed. Hanyuda et al. also used the median as cutoff, although counted in cells/mm^2^, rendering a higher count,^14^ whereas Wang et al. used the total number of CD3^+^ cells.^15^ Thus, the results may be difficult to compare, and future studies could benefit from using a pre-defined approach. Nevertheless, in this cohort, we found similar results when using cutoffs from classification and regression tree analyses.

In conclusion, obesity, measured as several anthropometric factors, was associated with risk of CRC with low density of tumor-infiltrating FoxP3^+^ T cells, but with high density of B cells and cytotoxic T cells in both sexes. There was a notable sex difference, despite the lack of a significant interaction with sex, in that obese women had a significant risk of CRC with high immune cell-specific PD-L1 expression, whereas obese men had a significant risk of CRC with low immune cell-specific PD-L1 expression. These findings suggest that both sex and anthropometric measurements may be important factors to take into account when assessing the potential prognostic or predictive value of immune biomarkers for improved treatment stratification of CRC patients.

## Material and methods

### Study cohort

The MDCS is a prospective population-based cohort,^32^ forming part of the EPIC cohort,^33^ that enrolled a total of 28 098 participants from a background population of 74 138; 11063 (39.4%) men and 17035 (60.6%) women, between 44 and 74 years of age. All participants completed the baseline examination, which included anthropometric measurements, a dietary assessment, and a questionnaire regarding lifestyle, health, and socio-demographic characteristics.

Cases were identified from the Swedish Cancer Registry up until 31 December 2007, and from The Southern Swedish Regional Tumor Registry for the period of 1 January – 31 December 2008. Median time from baseline until diagnosis was 8.6 (standard deviation [SD] = 4.3) years, and the median follow-up time in the entire cohort was 13.7 (SD = 3.2) years. There was a total number of 584 cases of incident CRC in the cohort. Eight tumors were re-classified as intramucosal (in situ) carcinoma and were not included as cases. Prevalent CRC was denoted in 66 cases, who were excluded from the analyses. Median age at diagnosis was 71 (range 50–86) years. Information on vital status and cause of death was obtained from the Swedish Cause of Death Registry up until 31 December 2013. Out of the 198 cases of rectal cancer, only 29% of the patients received neoadjuvant radiotherapy.

All tumors with available slides or paraffin blocks were histopathologically re-evaluated on hematoxylin and eosin-stained slides by a senior pathologist (KJ). Cases with an insufficient amount of tumor material were excluded, whereby a total number of 526 (90.1%) cases were available for tissue microarray (TMA) construction. For a few of the rectal cancers having received neo-adjuvant radiotherapy or in cases where the surgical specimen was missing (in total no more than five cases), diagnostic biopsies with an adequate amount of tumor tissue were included in the tissue microarrays. Representative and non-necrotic areas were marked, and TMAs were constructed as previously described.^34^ In brief, duplicate tissue cores (1 mm) were taken from non-necrotic areas in each primary tumor and mounted in a recipient block, using a semi-automated arraying device (TMArrayer, Pathology Devices, Westminister, MD, USA). Four µm sections from this block were subsequently cut using a microtome and mounted on glass slides.

### Immunohistochemical analyses

For immunohistochemical (IHC) analysis of CD68, 4 μm TMA-sections were pre-treated using ULTRA Cell Conditioning Solution 1, pH 8.5 (Ventana Medical Systems Inc., Tucson, AZ, USA), and then stained in a Ventana BenchMark stainer (Ventana Medical Systems Inc.) with a monoclonal anti-CD68 antibody (clone KP1, Dako, Glostrup, Denmark) diluted 1:1000. The immunohistochemical stainings and assessments of other immune cell subsets, PD-1, and PD-L1 have been described previously.^9–11^ In brief, for analyses of CD20^+^ B cells, and CD8^+^ and FoxP3^+^ T cells, the total number of stained cells (in the central tumor, the invasive margin, and in the peritumoral stroma) was used in the analyses, whereas immune cell-specific PD-L1 expression was annotated as the estimated percentage of stained cells and categorized as 0–9%, 10–49%, and 50–100% stained immune cells, and PD-L1 expression on tumor cells was annotated as the estimated percentage of stained cells and categorized as <1%, 1–4%, 5–9%, 10–49%, and 50–100% stained tumor cells. The number of CD8^+^ T cells was calculated by automated image analysis using the colocalization algorithm within the Halo image analysis software (Indica Labs, Corrales, NM). The number of CD20^+^ and FoxP3^+^ immune cells was evaluated manually. The PD-1 and PD-L1 stainings were evaluated independently by two observers (KJ and JB) blinded to clinical outcome, one being a board-certified pathologist (KJ). Discrepant cases were re-evaluated and discussed in order to reach consensus.

### Anthropometric measurements

At baseline examination, weight (multiples of 0.1 kg) and height (to the nearest 0.005 m) were measured by a trained nurse, and BMI was calculated as kg/m^2^. Waist circumference was measured at the midpoint between the lower ribs and the iliac crest, and the level of the greatest lateral extension was used for hip circumference, both measurements estimated to the nearest 0.01 m. The waist and hip circumferences of each participant were used to calculate WHR as an additional measure of fat distribution. Using a single frequency bio-impedance methodology (BIA 103, RLJ-systems, Detroit, MI, USA) with tetrapolar electrode placement and subjects in a supine position, lean body mass and fat mass were determined and served to calculate BFP. The BIA method has previously been validated in Swedish middle-aged and elderly adults.^35^ For analyses, anthropometric factors were assessed both as continuous variables and divided into quartiles, calculated from the full cohort, as well as separately in men and women.

### Ethics approval and consent to participate

All EU and national regulations and requirements for handling human samples have been fully complied with during the conduct of this project, that is, decision no. 1110/94/EC of the European Parliament and of the Council (OJL126 18,5,94), the Helsinki Declaration on ethical principles for medical research involving human subjects and the EU Council Convention on human rights and Biomedicine. Ethical permission for the MDCS (LU 90–51) and the present study (LU 530–2008) were obtained from the Ethics Committee at Lund University. Written informed consent has been obtained from each subject at study entry.

### Statistical analyses

Mann–Whitney U test and chi-square test were applied for assessment of the distribution of well-established and potential risk factors for CRC between cases and the rest of the cohort, and for assessment of the distribution of anthropometric and clinicopathological variables in cases across categories of low versus high immune cell density, and PD-L1 expression on immune cells and on tumor cells. Using the median values as cutoffs, cases were divided into groups of high (≥134, n = 250) and low (<134, n = 252) CD8^+^ T cell count; high (≥8, n = 262) and low (<8, n = 255) FoxP3^+^ cell count; and high (≥3, n = 266) and low (<3, n = 255) CD20^+^ B cell count. For PD-L1 expression on immune cells and tumor cells, cutoffs at 10% and 1% positive cells, respectively, were used, dividing cases into groups of high (n = 278) and low (n = 231) immune cell-specific PD-L1 expression, and high (n = 100) and low (n = 409) tumor cell-specific PD-L1 expression.

Cox proportional hazard models were used to assess the impact of different anthropometric factors on risk of CRC defined by low and high expression of different tumor-infiltrating immune cell markers, using the median value as cutoff. In the multivariable Cox regression analysis, potential confounders were included, i.e. age (years), smoking habits (yes regularly, yes occasionally, former smoker, never smoker), alcohol consumption (grams/day), and educational level (≤8 years, 9–10 years or 11–13 years of education, and university degree). Anthropometric factors were examined as continuous variables as well as quartiles, and trend was calculated as linear trend over quartiles. Missing category was not included in the trend analysis. The proportional hazards assumption was tested using Cox regression with a time-dependent covariate analysis, whereby the proportional hazard assumption was considered to be satisfied when the factor x time interaction was non-significant.

A case-to-case analysis examined the heterogeneity of immune cell densities regarding their associations to anthropometric factors, using an unconditional logistic regression model. The potential interaction between each anthropometric factor and sex was tested with the covariates age, sex, anthropometric factor, and anthropometric factor × sex, and also repeated with the fully adjusted model.

All calculations were performed using SPSS version 24.0 (SPSS Inc, Chicago, IL). All statistical tests were two-sided and *p*-values <0.05 were considered statistically significant. Nominal *p*-values are presented without adjustment for multiple testing, as this is an exploratory study.^36^

## Abbreviations

BFP: body fat percentage
BMI: body mass index
CI: confidence interval
CRC: colorectal cancer
dMMR: deficient mismatch repair
EPIC: European Prospective Investigation into Cancer and Nutrition
HR: hazard ratio
IHC: immunohistochemical
MDCS: Malmö Diet and Cancer Study
MSI: microsatellite instability
MSS: microsatellite stable
PD-1: programmed cell death protein 1
PD-L1: programmed cell death protein ligand 1
PDL1/IC: programmed cell death protein ligand 1 on immune cells
PDL1/TC: programmed cell death protein ligand 1 on tumor cells
pMMR: proficient mismatch repair
SD: standard deviation
TMA: tissue microarray
WHR: waist-hip ratio
