# Supplementary material for: Pre-diagnostic anthropometry, sex, and risk of colorectal cancer according to tumor immune cell composition
Source: Oncoimmunology. 2019 Sep 19;8(12):e1664275. doi: 10.1080/2162402X.2019.1664275 (PMC6844316; doi:10.1080/2162402X.2019.1664275)
Supplement: Supplemental Material [file koni-08-12-1664275-s001.docx]

**Supplementary Table 2: Distribution of risk factors in EPIC cases and non-EPIC cases, respectively**

| **Factor** | **EPIC**  **(n = 584)** | **Non-EPIC**  **(n = 42)** | ***p*** |
| --- | --- | --- | --- |
| **Age at baseline** | 63.1 (6.8) | 62.5 (6.1) | *0.879* |
| **Height, cm** | 169.0 (8.8) | 165.0 (10.3) | *0.238* |
| **Weight, kg** | 75.0 (14.2) | 73.0 (13.7) | *0.504* |
| **Body-mass index, kg/m^2^** | 26.2 (4.0) | 25.7 (3.6) | *0.955* |
| **Waist circumference, cm** | 87.0 (13.7) | 91.0 (12.9) | *0.801* |
| **Hip circumference, cm** | 100.0 (9.0) | 99 (7.6) | *0.578* |
| **Waist-hip-ratio** | 0.86 (0.10) | 0.87 (0.09) | *0.272* |
| **Body-fat percentage, %** | 27.0 (7.1) | 28.0 (6.9) | *0.618* |
| **Alcohol consumption, g/day** | 6.3 (14.2) | 10.5 (-) | *0.605* |
| **Smoking status** |  |  | *0.560* |
| Regularly | 124 (21.2) | 1 (14.3) |  |
| Occasionally | 20 (3.4) | 0 (0.0) |  |
| Former smoker | 232 (39.7) | 3 (42.9) |  |
| Never smoked | 208 (35.6) | 3 (42.9) |  |
| **Educational** |  |  | *0.436* |
| ≤8 years | 292 (50.3) | 3 (57.1) |  |
| 9-10 years | 130 (22.4) | 2 (28.6) |  |
| 11-13 years | 96 (16.5) | 1 (14.3) |  |
| University degree | 63 (10.8) | 0 (0.0) |  |
| **T-stage** |  |  | *0.128* |
| 1 | 51 (9.8) | 0 (0.0) |  |
| 2 | 62 (12.0) | 3 (9.7) |  |
| 3 | 323 (62.4) | 23 (74.2) |  |
| 4 | 82 (15.8) | 5 (16.1) |  |
| **N-stage** |  |  | *0.941* |
| 0 | 286 (57.9) | 19 (61.3) |  |
| 1 | 124 (25.1) | 6 (19.4) |  |
| 2 | 84 (17.0) | 6 (19.4) |  |
| **M-stage** |  |  | *0.784* |
| 0 | 444 (79.3) | 31 (77.5) |  |
| 1 | 116 (20.7) | 9 (22.5) |  |
| **Differentiation grade** |  |  | *0.782* |
| High/intermediate | 421 (77.4) | 27 (79.4) |  |
| Low | 123 (22.6) | 7 (20.6) |  |
| **MSI** |  |  | *0.097* |
| MSS | 423 (86.5) | 20 (74.1) |  |
| MSI | 66 (13.5) | 7 (25.9) |  |
| **KRAS** |  |  | *0.142* |
| Wild-type | 395 (80.3) | 20 (69.0) |  |
| Mutated | 97 (19.7) | 9 (31.0) |  |
| **BRAF** |  |  | *0.921* |
| Wild-type | 345 (69.8) | 20 (69.0) |  |
| Mutated | 149 (30.2) | 9 (31.0) |  |
| **Tumour location** |  |  | *0.735* |
| Right colon | 205 (36.2) | 16 (39.0) |  |
| Left colon | 143 (25.3) | 10 (24.4) |  |
| Rectum | 218 (38.5) | 15 (36.6) |  |

Median [standard deviation (SD)] presented for continuous variables.
