# Supplementary material for: Pre-diagnostic anthropometry, sex, and risk of colorectal cancer according to tumor immune cell composition
Source: Oncoimmunology. 2019 Sep 19;8(12):e1664275. doi: 10.1080/2162402X.2019.1664275 (PMC6844316; doi:10.1080/2162402X.2019.1664275)
Supplement: Supplemental Material [file koni-08-12-1664275-s002.docx]

**Supplementary Table 1: Distribution of risk factors in men and women, respectively**

| **Factor** | **Men**  **(n = 280)** | **Women**  **(n = 304)** | ***p*** |
| --- | --- | --- | --- |
| **Age at baseline** | 62.7 (6.7) | 63.6 (6.9) | *0.239* |
| **Alcohol consumption, g/day** | 11.8 (17.4) | 3.8 (4.9) | *<0.001* |
| **Smoking status** |  |  | *0.108* |
| Regularly | 54 (19.0) | 71 (23.1) |  |
| Occasionally | 12 (4.2) | 8 (2.6) |  |
| Former smoker | 148 (52.1) | 87 (28.3) |  |
| Never smoked | 70 (24.6) | 141 (45.9) |  |
| **Educational** |  |  | *0.148* |
| ≤8 years | 141 (49.6) | 155 (51.0) |  |
| 9-10 years | 57 (20.1) | 75 (24.7) |  |
| 11-13 years | 48 (16.9) | 49 (16.1) |  |
| University degree | 38 (13.4) | 25 (8.2) |  |
| **T-stage** |  |  | *0.652* |
| 1 | 18 (6.9) | 33 (11.5) |  |
| 2 | 35 (13.4) | 30 (10.5) |  |
| 3 | 182 (65.6) | 174 (60.6) |  |
| 4 | 37 (14.1) | 50 (17.4) |  |
| **N-stage** |  |  | *0.144* |
| 0 | 143 (57.0) | 162 (59.1) |  |
| 1 | 55 (21.9) | 75 (27.4) |  |
| 2 | 53 (21.1) | 37 (13.5) |  |
| **M-stage** |  |  | *0.546* |
| 0 | 225 (78.1) | 250 (80.1) |  |
| 1 | 63 (21.9) | 62 (19.9) |  |
| **Differentiation grade** |  |  | *0.187* |
| High/intermediate | 219 (79.9) | 229 (75.3) |  |
| Low | 55 (20.1) | 75 (24.7) |  |
| **MSI** |  |  | *0.959* |
| MSS | 189 (80.4) | 204 (86.1) |  |
| MSI | 31 (14.1) | 33 (13.9) |  |
| **KRAS** |  |  | *0.569* |
| Wild-type | 180 (81.4) | 192 (79.3) |  |
| Mutated | 41 (18.6) | 50 (20.7) |  |
| **BRAF** |  |  | *0.946* |
| Wild-type | 152 (68.8) | 166 (68.3) |  |
| Mutated | 69 (31.2) | 77 (31.7) |  |
| **Tumour location** |  |  | *0.151* |
| Right colon | 97 (33.6) | 124 (39.0) |  |
| Left colon | 74 (25.6) | 79 (24.8) |  |
| Rectum | 118 (40.8) | 115 (36.2) |  |

Median [standard deviation (SD)] presented for continuous variables.
